# A role for tau in learning, memory and synaptic plasticity

**DOI:** 10.1038/s41598-018-21596-3

**Published:** 2018-02-16

**Authors:** Fabrizio Biundo, Dolores Del Prete, Hong Zhang, Ottavio Arancio, Luciano D’Adamio

**Affiliations:** 1 Department of Microbiology & Immunology, Albert Einstein College of Medicine, 1300 Morris Park Avenue, Bronx, NY 10461 USA; 2 Department of Pathology and Cell Biology and Taub Institute for Research on Alzheimer’s Disease and the Aging Brain, Columbia University, 630 168 St., New York, NY 10032 USA; 3 Department of Pharmacology, Physiology & Neuroscience New Jersey Medical School, Brain Health Institute, Rutgers, The State University of New Jersey, 185 South Orange Ave, Newark, NJ 07103 USA

## Abstract

Tau plays a pivotal role in the pathogenesis of neurodegenerative disorders: mutations in the gene encoding for tau (*MAPT*) are linked to Fronto-temporal Dementia (FTD) and hyper-phosphorylated aggregates of tau forming neurofibrillary tangles (NFTs) that constitute a pathological hallmark of Alzheimer disease (AD) and FTD. Accordingly, tau is a favored therapeutic target for the treatment of these diseases. Given the criticality of tau to dementia’s pathogenesis and therapy, it is important to understand the physiological function of tau in the central nervous system. Analysis of *Mapt* knock out (*Mapt*^−/−^) mice has yielded inconsistent results. Some studies have shown that tau deletion does not alter memory while others have described synaptic plasticity and memory alterations in *Mapt*^−/−^ mice. To help clarifying these contrasting results, we analyzed a distinct *Mapt*^−/−^ model on a B6129PF3/J genetic background. We found that tau deletion leads to aging-dependent short-term memory deficits, hyperactivity and synaptic plasticity defects. In contrast, *Mapt*^+/−^ mice only showed a mild short memory deficit in the novel object recognition task. Thus, while tau is important for normal neuronal functions underlying learning and memory, partial reduction of tau expression may have fractional deleterious effects.

## Introduction

Tau is a microtubule-associated protein involved in neurodegenerative disorders. Hyper-phosphorylated aggregates of tau are the main components of NFTs, which, together with amyloid plaques and neuronal loss, constitute the primary pathological hallmarks of AD. In addition, several *MAPT* mutations are genetically linked to FTD^1–9^. Finally, in recent years extracellular soluble tau oligomers have been recognized as a possible cause of memory loss and synaptic dysfunction^10–14^.

The mechanisms underlying the pathogenic role of tau in AD have been the subject of many studies. AD pathogenesis is linked to the Amyloid-β Precursor Protein (APP) and Aβ^15–21^, a peptide derived from APP processing. Mutations in APP cause familial forms of AD (FAD) and overexpression of FAD APP mutants in mice has been routinely used to model AD. FAD APP mutant mice develop cognitive and synaptic plasticity deficits. These deficits can be prevented through the ablation of tau expression^22–25^, leading to the hypothesis that tau is required for Aβ-induced synaptic dysfunction and memory deficits.

Clinical trials targeting Aβ have yielded disappointing results and consequently, the focus of AD therapeutic approaches has turned to the elimination of toxic tau forms through the use of anti-tau antibodies or antisense oligonucleotides^26–35^. A byproduct of these therapies is the reduction of total tau levels. Thus, it is important to determine the normal function of tau and whether reducing tau levels may trigger unwanted deleterious consequences.

Various functions of tau have been proposed^4^, however it has been challenging to confirm them in *Mapt*^−/−^ mice, probably due to compensatory mechanisms. For example, other microtubule-associated proteins may compensate for the proposed microtubule stabilization and the axonal transport functions of tau in *Mapt*^−/−^ mice^24,36,37^. Tau also participates in protein trafficking, as loss of tau changes the distribution of several proteins, including Fyn and APP^22,38^. *Mapt*^−/−^ mice display compromised synaptic function, evidenced by impaired long-term potentiation (LTP)^39^ -a long term synaptic plasticity mechanism thought to underlie memory- and long-term depression (LTD)^40,41^.

While some studies have revealed no cognitive deficits in *Mapt*^−/−^ mice^22,23,38,42^, others have reported some deficits. These include impaired contextual and cued fear condition at 6 months of age^39,43^ and impaired Morris water maze performance at 20, but not 12, months of age^44^. These differences in cognitive impairments may depend on the mouse genetic background^45^, but also the type and conditions of behavioral/memory tests performed, as well as variations in diet and housing conditions. Another important variable is the *Mapt*^−/−^ model used, as four different *Mapt*^−/−^ mice have been generated^46–48^.

We have analyzed the *Mapt*^−/−^ model developed by Tucker^48^ and we have tested *Mapt*^+/−^, *Mapt*^−/−^ and WT littermates on a hybrid B6129PF3/J genetic background. Hybrid display better memory scores than their parental strains, a phenomenon known as hybrid vigor^49,50^. Here, we report that *Mapt*^−/−^ mice develop aging-dependent memory and learning impairments and deficits in LTP. In contrast, *Mapt*^+/−^ mice showed a mild short memory deficit. These results suggest that tau plays a role in learning and memory; however, a partial reduction of tau protein levels may cause minor cognitive impairments. These findings have valuable implications regarding our understanding of tau biology and of tau as a drug target.

## Results

**Mild short-term memory deficits and increased locomotor activity in six-seven-month-old** ***Mapt***^**−/−**^ **mice**. To determine the impact of *Mapt* deletion on anxiety-like behavior, locomotor activity and short-term memory, six-seven month-old WT, *Mapt*^+/−^ and *Mapt*^−/−^ B6129PF3/J mice (see Material and methods for details concerning the generation of these animals) were subjected to the following behavioral tasks: 1) the elevated zero maze, which tests anxiety-like behavior; 2) the two-trial Y maze, to analyze locomotor activity and short-term spatial recognition memory; 3) open field, a test to determine locomotion, exploratory activities, and anxiety; 4) novel object recognition, a short-term memory test.

**Figure 1:**
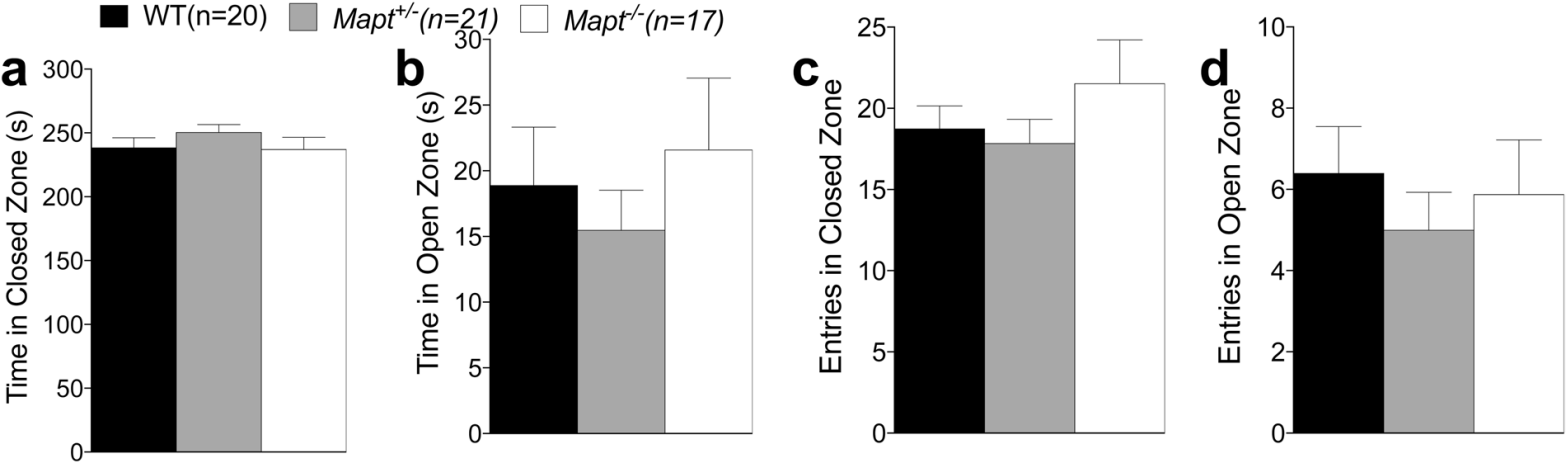
The elevated zero maze revealed no anxiety deficit in both *Mapt*^+/−^ and *Mapt*^−/−^ mice at the age of six months. The amount of time spent in the open and closed zones and the number of entries into the open and closed zones of the elevated zero maze during the 5-min testing period were analyzed (**a–d**). One-way ANOVA revealed no significant effect of genotype for all the measures.

In the elevated zero maze no effect of genotype was detected for time spent in the open and closed zones [one-way ANOVA, F (2, 55) = 0.5070, P = 0.6051; F (2, 55) = 0.9461, P = 0.3945], and number of entries into the open and closed zones [one-way ANOVA, F (2, 55) = 1.024, P = 0.3659; F (2, 55) = 0.4186, P = 0.6601], suggesting that *Mapt* deletion does not cause anxiety-like behavior (Fig. 1a–d).

**Figure 2:**
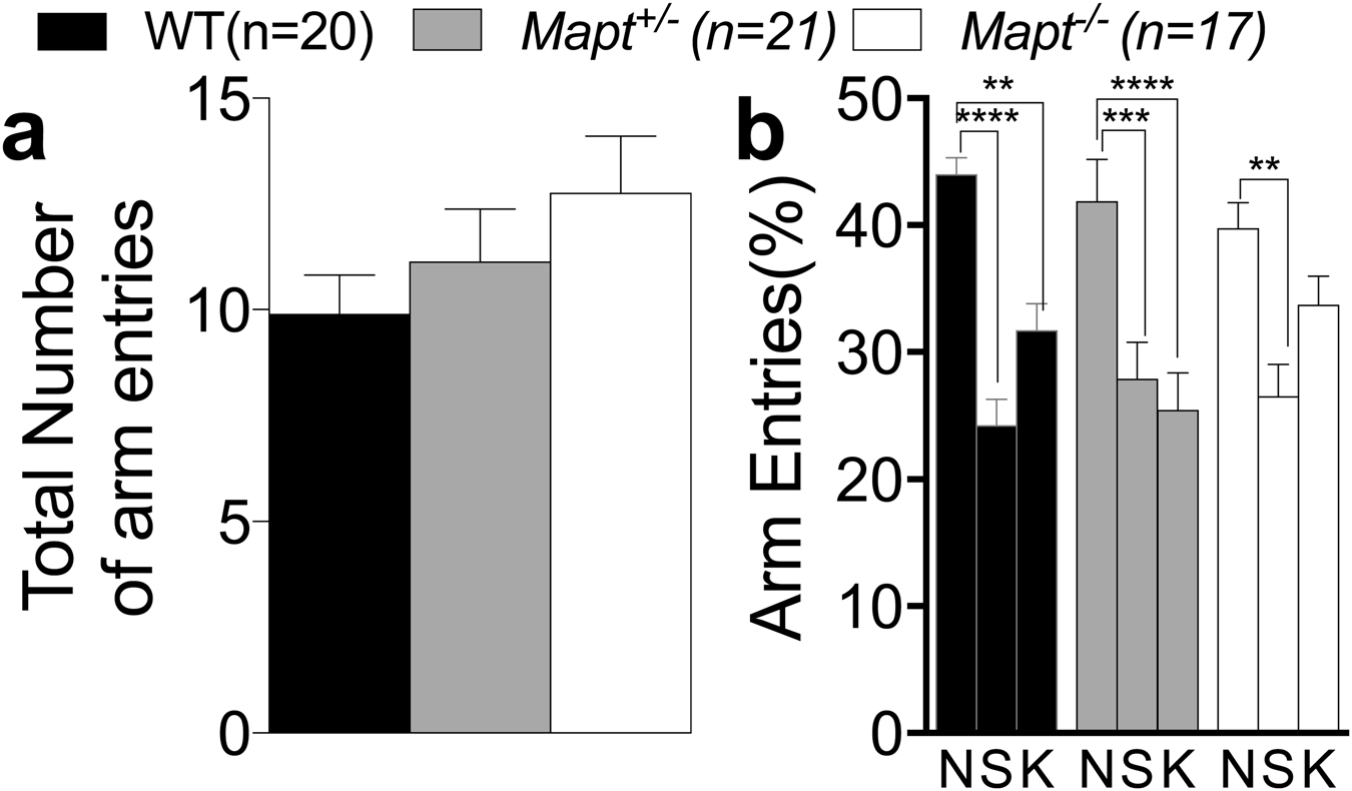
The two trial Y-maze task showed a mild deficit of short-term spatial recognition memory in *Mapt*^−/−^ mice at the age of 6 months. Mice were analyzed at 6 months of age. (**a**) Total number of arm entries during trial 2. (**b**) Percentage of entries into the novel (N), start (S) and known (K) arms. Data are expressed as means ± S.E.M.: **P < 0.01; ***P < 0.001, ****P < 0.0001 (Dunnett’s multiple comparisons test).

In the two-trial Y-maze task, locomotor activity was assessed as total number of entries into the three arms during the second trial. The differences among genotypes were not statistically significant [one-way ANOVA, F (2, 55) = 1.430, P = 0.2480, Fig. 2a]. Next, we calculated the number of entries (expressed as percentage) into each arm during trial 2, to evaluate mice’s tendency of exploring the new arm (N) versus the other two familiar arms (K) explored during trial 1. As shown in Fig. 2b, two-way ANOVA detected a main effect for arm [F (2, 110) = 25.55, P < 0.0001)]. Dunnett’s multiple comparisons test within each genotype showed a major tendency to enter into the novel arm by WT and *Mapt*^+/−^ mice, while *Mapt*^−/−^ did not significantly discriminate the K arm from the N arm suggesting that the deletion of both *Mapt* alleles mildly impairs the short-term spatial recognition memory at this age.

**Figure 3:**
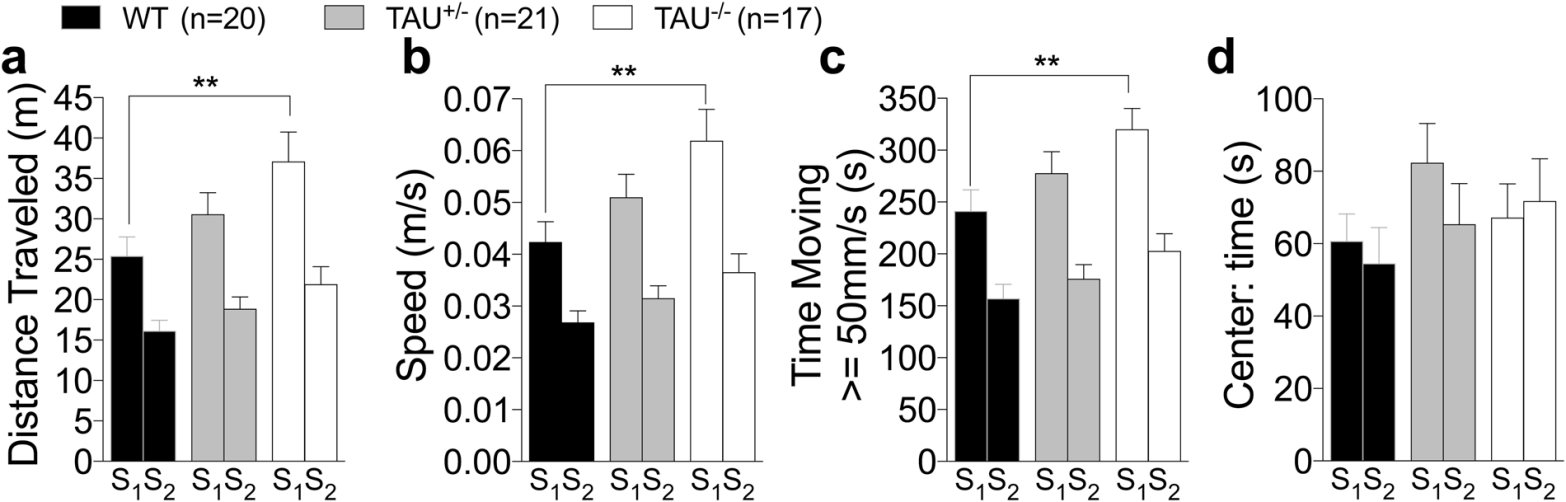
Increased locomotor activity of *Mapt*^−/−^ mice at the age of 7 months in the open field test. (**a**) Mean distance traveled: *Mapt*^−/−^ mice traveled significantly longer distances than WT animals during the first session. (**b**) Mean speed: *Mapt*^−/−^ mice traveled at a significantly higher speed than littermate control mice during the first session. (**c)** Time spent traveling at speed greater than 50 mm/s. *Mapt*^−/−^ mice spent more time moving > =50 mm/s than WT mice during the first session. (**d**) Mean time spent in the center of the open field is not significantly different among the three genotypes. Data are expressed as means ± S.E.M.: **P < 0.01, Tukey’s multiple comparisons test.

In the open field test, distance travelled, speed, and time moving at = >50 mm/s are indexes of locomotor activity. Two-way ANOVA showed an effect of genotype on locomotor activity [Two-way ANOVA distance: F (2, 55) = 3.952, P = 0.049; speed: F (2, 55) = 3.969, P = 0.0245; time moving > =50 mm/s: F (2, 55) = 3.326, P = 0.0433, Fig. 3a–c], with *Mapt*^−/−^mice being significantly more active than WT mice in exploring the field during the first session (Tukey’s multiple comparisons test). All genotypes are less active on the second session indicating a similar habituation to the field during the second day. *Mapt*^+/−^ mice and *Mapt*^−/−^animals spent the same time in the center as WT littermates in both sessions [F (2, 55) = 0.8957, P = 0.4142, Fig. 3d] confirming that *Mapt* deletion produces no anxiety-like behavior.

**Figure 4:**
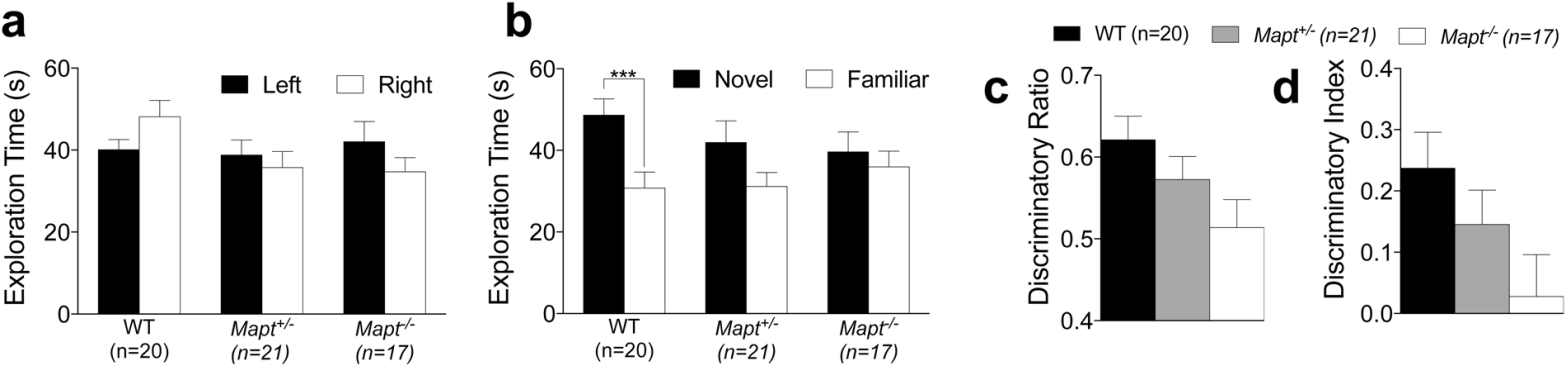
Novel object recognition test shows that 7-month-old *Mapt*^−/−^ mice present a mild short-term memory deficit. Data are expressed as means ± S.E.M. (**a**) Time spent during the exploration of two familiar objects. (**b**) Time spent exploring the familiar and novel objects. While WT mice explore more the novel object (***P = 0.0002, Sidak’s multiple comparisons test), *Mapt*^+/−^ and *Mapt*^−/−^ mice spent comparable amount of time exploring the two objects. Discriminatory ratio and discriminatory index are shown in (**c**) and (**d**).

In the novel object recognition test, the animals were first exposed to two identical familiar objects for ten minutes. Mice of all genotypes showed no object preference (Fig. 4a). After a four hours interval, mice were allowed to explore for ten minutes one of the familiar objects paired to a novel one. ANOVA found that the genotype factor did not statistically have an effect, while the object factor did [F (2, 55) = 0.2145, P = 0.8076; F (1, 55) = 16.13, P = 0.0002]. Post hoc multiple comparisons (Sidak’s) revealed that WT mice explored the novel object significantly more than the familiar one (P = 0.0002), while *Mapt*^+/−^ and *Mapt*^−/−^ did not show any significant difference in exploring the two objects (Fig. 4b). Ordinary one-way ANOVA analysis of discriminatory ratio (DR) and discriminatory index (DI) approached, but did not reach, significance (DR: F (2, 55) = 0.361, P = 0.0549; DI: F (2.55) = 0.2894, P = 0.0638) (Fig. 4c and d). Overall the data suggest that *Mapt*^+/−^ and *Mapt*^−/−^ may have a deficit in preference for the novel object when compared to their control.

### Short-term memory deficits of *Mapt*^−/−^ mice exacerbate with aging

**Figure 5:**
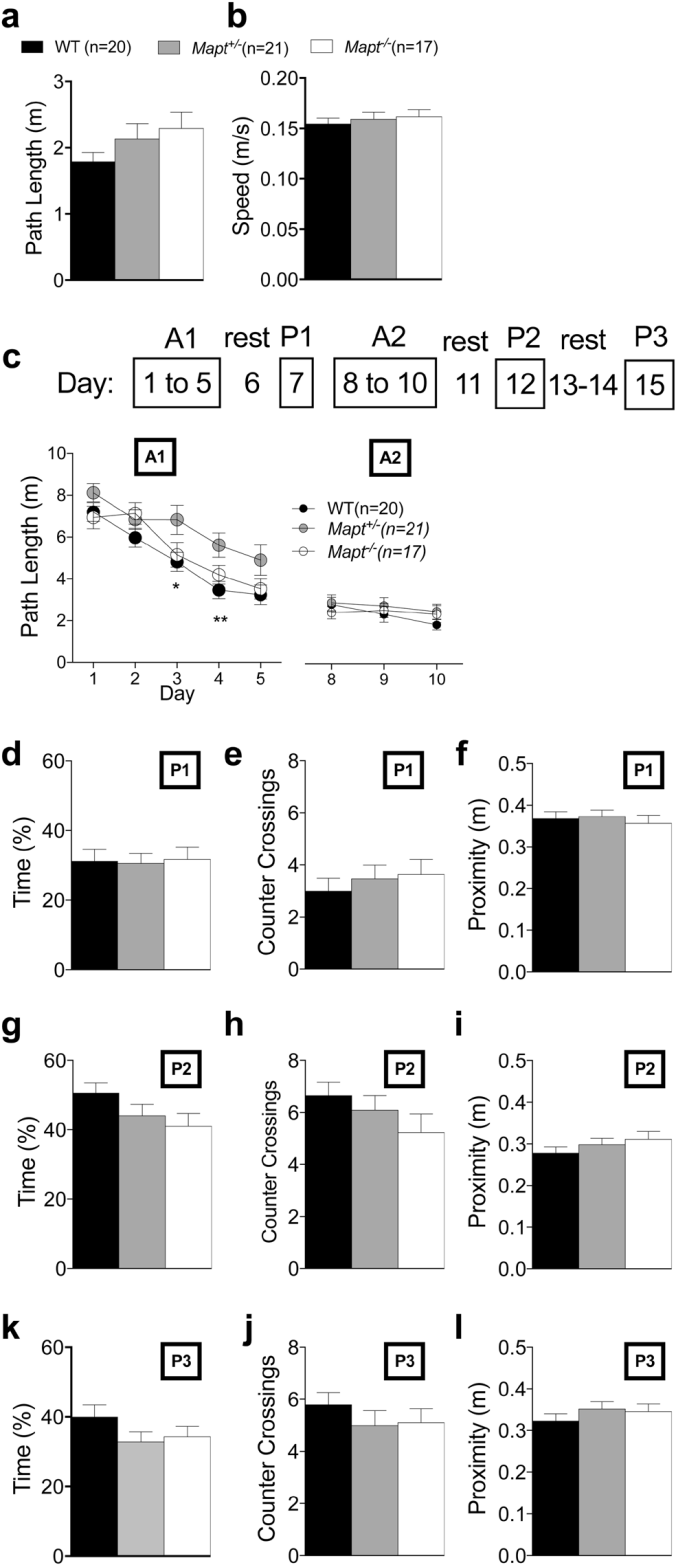
The visible platform and the Morris water maze tests show no visual, motivational spatial learning and retention memory deficit in 10-month-old *Mapt*^−/−^ mice. Visual task: path length swam to reach the visible platform (**a**) and mean speed with which mice swam to reach the visible platform (**b**) revealed no significant effect of genotype (one-way ANOVA). (**c**) The schematic illustration of the Morris water maze task is represented above the mean path lengths for the A1 and A2 sessions. (**d**–**f**) Time spent in the target quadrant during the P1, P2 and P3 trials; (**g**–**h**) Counter crossings in the target platform during the P1, P2 and P3 trials; (**k**–**l**) Proximity to the target platform during the P1, P2 and P3 trials. Data are expressed as means ± S.E.M, *P < 0.05, **P < 0.01, Tukey’s multiple comparisons test.

To further evaluate the impact of tau deletion on spatial learning and retention memory, ten-eleven-month-old were assessed in the classic version of water maze developed by Richard Morris^51^. The cued learning task in which the platform was made visible by a flag did not show any difference between control and mutant groups in either distance covered [F (2, 55) = 1.520, P = 0.2277] or swim velocity [F (2, 55) = 0.3216, P = 0.7263] (Fig. 5a,b).

These animals were suitable to study spatial learning and memory using the Morris water maze since none of the experimental groups had motor, visual or motivational alterations. The experiment was carried out as follows: 1) five-days long acquisition of the hidden platform task (referred to as A1); 2) probe trial conducted 2 days later (P1, at day 7); 3) second 3-days long acquisition trial (A2, days 8–10); 4) probe trial run 2 days later (P2, day 12); 5) last probe trial conducted after an additional 3 days (P3, day 15) (see scheme in above Fig. 5c). Figure 5c shows the distance covered to reach the hidden platform in the A1 and A2 sessions. As detected by ANOVA, the genotype produced a significant effect in learning the location of the platform over the five-day period of time during the A1 session [F (2, 55) = 5.078; P = 0.0095)]. Tukey’s test detected a difference with *Mapt*^+/−^ covering a longer path than control group during day 3 and day 4 (P < 0.05, P < 0.01). No significant results were found during the A2 session [F (2, 110) = 2.249; P = 0.1103, F (2, 55) = 0.5569; P = 0.5762]. One-way ANOVA analysis of the P1, P2 and P3 trials revealed no significance when the time employed in the target quadrant [P1 trial, F (2, 55) = 0.03002, P = 0.974, Fig. 5d; P2 trial, F (2, 55) = 2.142, P = 0.1271, Fig. 5e; P3 trial, F (2, 55) = 1.533; P = 0.22249, Fig. 5f], the number of target platform crossings [P1 trial, F (2, 55) = 0.4067, P = 0.6678, Fig. 5g; P2 trial, F (2, 55) = 1.386, P = 0.2588, Fig. 5h; P3 trial, F (2, 55) = 0.7089; P = 0.4966, Fig. 5i] and the proximity to the area in which the platform was originally located [P1 trial, F (2, 55) = 0.2506, P = 0.7792, Fig. 4k; P2 trial, F (2, 55) = 1.134, *p* = 0.3291, Fig. 5j; P3 trial, F (2, 55) = 0.8308, P = 0.4411, Fig. 5l] were considered. Overall, these data suggest that tau deletion does not appreciably impact spatial learning and memory at this age.

**Figure 6:**
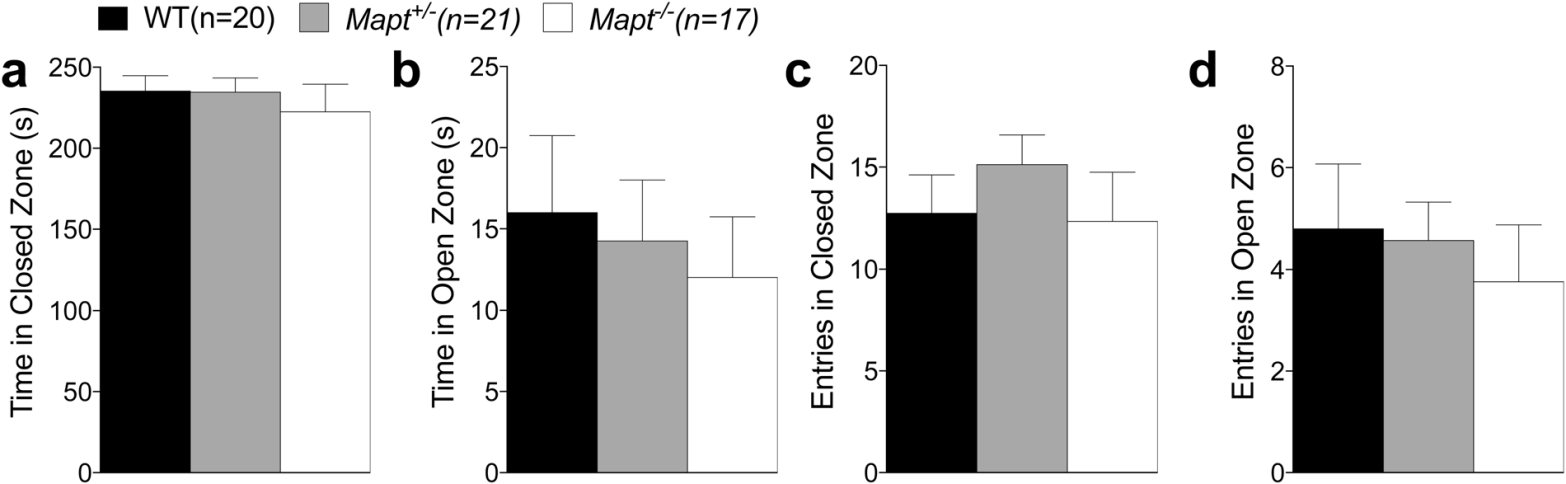
The elevated zero maze revealed no anxiety deficit in both *Mapt*^+/−^ and *Mapt*^−/−^ mice at the age of twelve months. The amount of time spent in the open and closed zones and the number of entries into the open and closed zones of the elevated zero maze during the 5-min testing period were analyzed (**a–d**). One-way ANOVA revealed no significant effect of genotype for all the measures.

**Figure 7:**
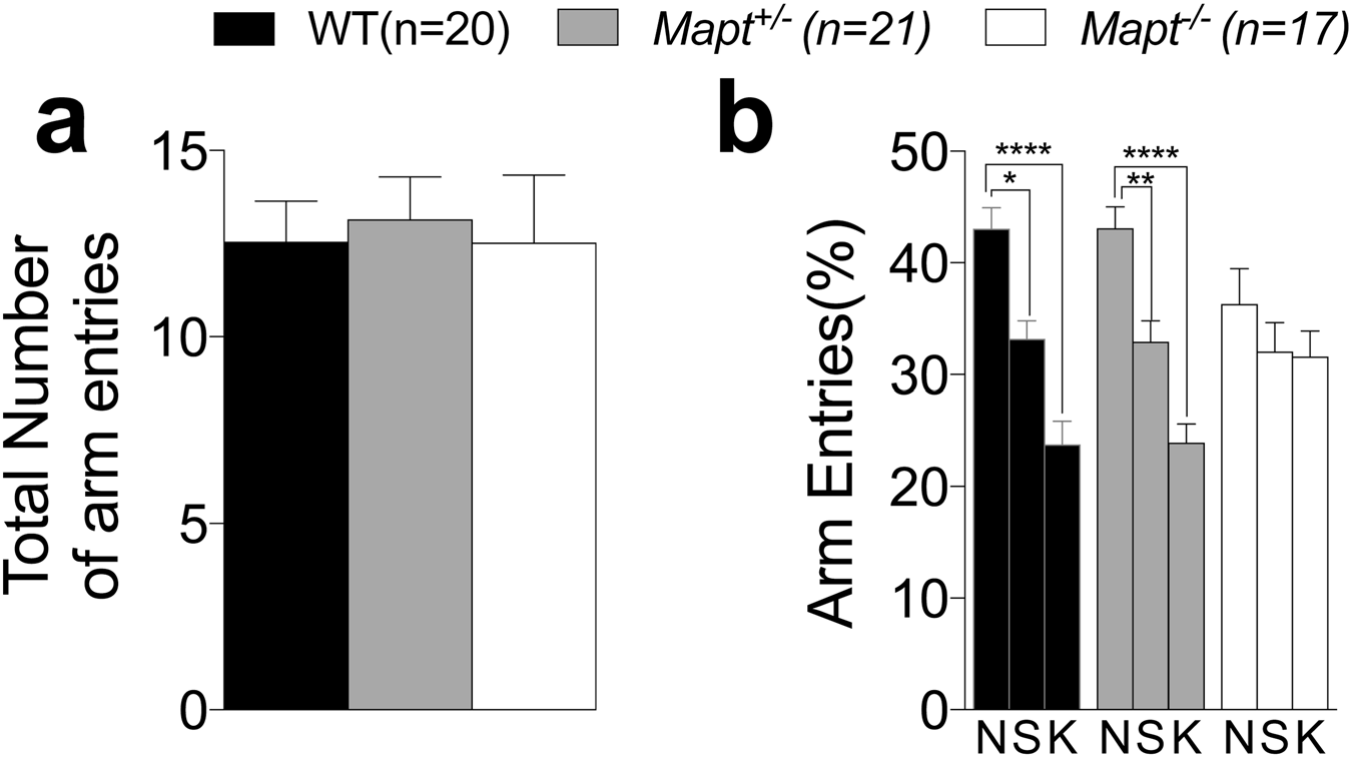
Short-term spatial recognition memory deficits of *Mapt*^−/−^ mice in the two trial Y-maze task exacerbate with aging. Mice were analyzed at 12 months of age. (**a**) Total number of arm entries during trial 2. (**b**) Percentage of entries into the novel (N), start (S) and known (K) arms. Data are expressed as means ± S.E.M; *P < 0.05, **P < 0.01, ****P < 0.0001, Dunnett’s multiple comparisons test.

To monitor the effect of tau deletion on memory and anxiety over time, mice were further tested in the elevated zero maze, Y maze and Open Field at the age of twelve months. In line with what observed at six months of age (Fig. 1a), one-way ANOVA analysis of the elevated zero maze test found no significant main effect for genotype for entries and time spent into the zones [F (2, 55) = 0.2212; P = 0.8023, F (2, 55) = 0.3623, P = 0.6977; F (2, 55) = 0.66, P = 0.5299; F (2, 55) = 0.2471, P = 7480 Fig. 6a–d], confirming that *Mapt* deletion does not cause anxiety behavior alterations. On the Y maze task, all three groups exhibited the same level of activity in exploring the three arms [one-way ANOVA, F (2, 55) = 0.07045, P = 0.9321, Fig. 7a]. When the exploration of the three arm was assesses as entries’ percent, two-way ANOVA did not detect any significance for genotype [F (2, 55) = 0.9018; P = 0.4118], but a significant effect was found when the interaction between arm factor and genotype factor was calculated [F (4, 110) = 2.503; P = 0.0464. Dunnett’s multiple comparisons test within the arm factor showed that WT and *Mapt*^+/−^ mice showed a major exploration of the novel arm compared to the known ones. In contrast, *Mapt*^−/−^ mice could not discriminate the N arm from the S and K arms, suggesting that the short-memory deficit previously observed at six months of age (Fig. 2b) worsen in an aging-dependent manner in mice that do not express tau.

**Figure 8:**
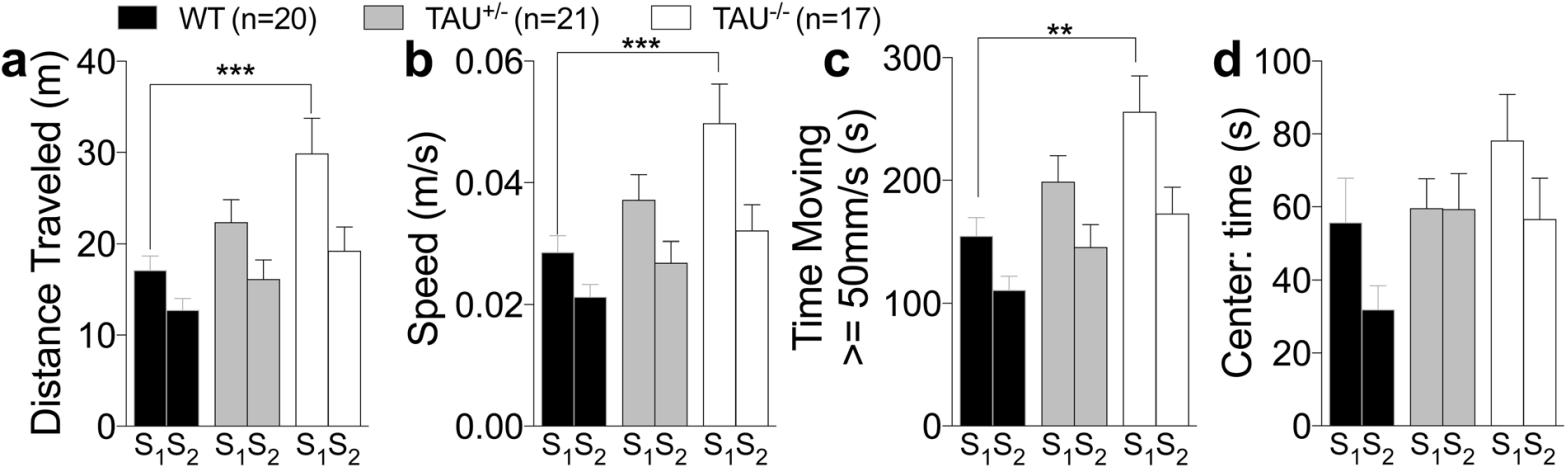
Increased locomotor activity of *Mapt*^−/−^ mice at the age of 11 months in the open field test. (**a**) Mean distance traveled: *Mapt*^−/−^ mice traveled significantly longer distances than WT animals during the first session. (**b**) Mean speed: *Mapt*^−/−^ mice traveled at a significantly higher speed than littermate control mice during the first session. (**c**) Time spent traveling at speed greater than 50 mm/s. *Mapt*^−/−^ mice spent more time moving > =50 mm/s than WT mice during the first session. (**d**) Mean time spent in the center of the open field is not significantly different among the three genotypes. Data are expressed as means ± S.E.M: **P < 0.01, ***P < 0.001, Tukey’s multiple comparisons test.

Consistent with what observed at seven months of age (Fig. 3a–c), open field data showed that *Mapt*^−/−^ mice exhibited increased locomotor activity compared to WT mice. Two-way ANOVA analysis of distance, speed and moving = >50 mm/s highlighted a significant result when the genotype factor was taken into account [distance, F (2, 55) = 4.877, P = 0.0112; speed, F (2, 55) = 4.847, P = 0.0115; moving, F (2, 55) = 5.178, P = 0.0087] (Fig. 8a–c). Tukey’s multiple comparisons test showed a significant difference between *Mapt*^−/−^ mice and WT mice in all three indexes during the first session. All groups spent similar amount of time in the central area of the field [two-way ANOVA, genotype, F (2, 55) = 1.891; P = 0.1606], further indicating that *Mapt* deletion does not cause anxiety-like behavior (Fig. 8d).

### Working memory and contextual memory deficits in eighteen-month old *Mapt*^−/−^ mice

**Figure 9:**
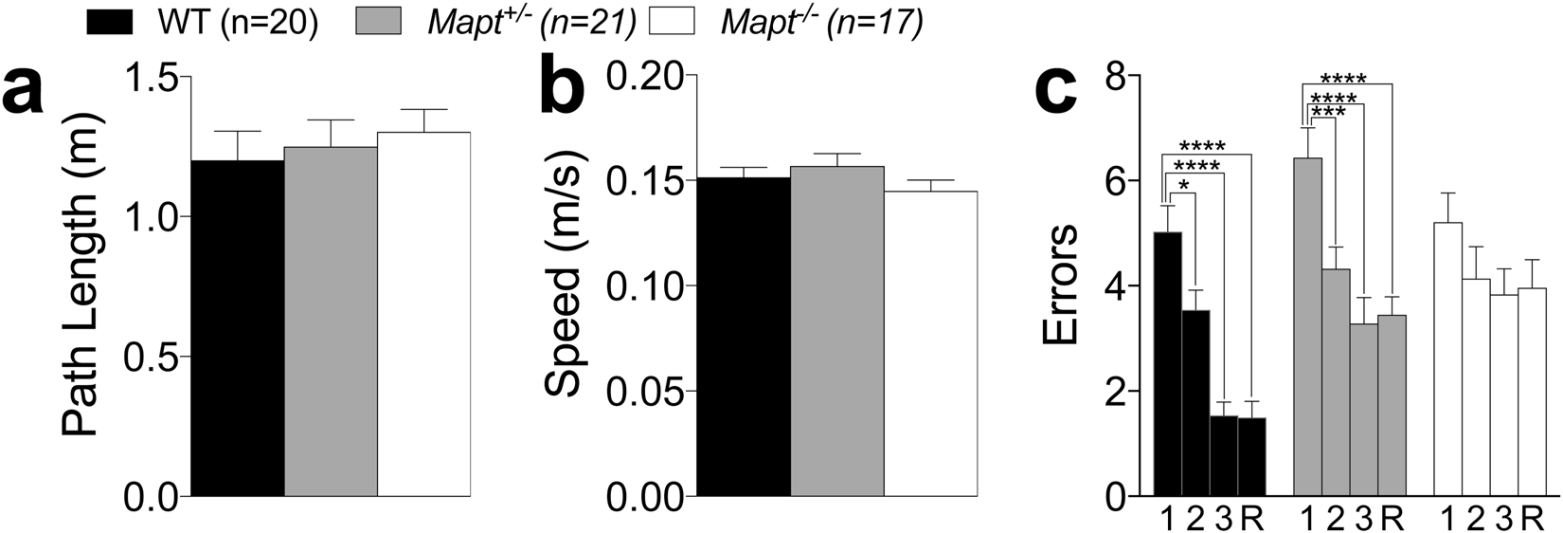
*Mapt*^−/−^ mice exhibited a dysfunction in working memory at the age of 16 months in the 6-arm radial water maze. Visual task: path length swam to reach the visible platform (**a**) and mean speed with which mice swam to reach the visible platform (**b**) revealed no significant effect of genotype (one-way ANOVA). (**c**) average number of errors in finding the platform across four trials. Data are expressed as means ± S.E.M: *P < 0.05, ***P < 0.001, ****P < 0.0001, Dunnett’s multiple comparisons test.

The experimental groups were assayed in the 6-radial arm water maze (RAWM) for working memory at the age of sixteen months. A visual task was performed before this test. No main genotype’s effect in both path length and speed covered to reach the visible platform [F (2, 55) = 0.2645, P = 0.7685; F (2, 55) = 1.241, P = 0.2969] was detected, indicating no visual, motor or motivational deficits in 15 month-old mutant mice (Fig. 9a,b).

The platform location was changed daily and mice were given four trials to locate the platform hidden at the end of one of the six arms. A significant effect was found for all the statistical factors: genotype [F (2, 55) = 8.276; P = 0.0007, trial [F (3, 165) = 27.97; P < 0.0001], and their interaction [F (6, 165) = 2.218; P = 0.0438]. Tukey’s multiple comparisons test showed that, while WT and *Mapt*^+/−^ mice performed significantly better between trials and remembered the locations of the platform at the retention trial, *Mapt*^−/−^ mice did not (Fig. 9c).

**Figure 10:**
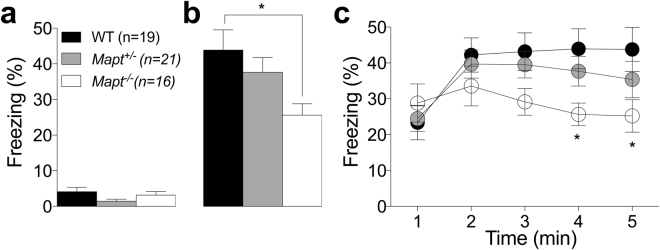
Fear conditioning paradigm conducted at the age of 18 months show contextual memory deficit for *Mapt*^−/−^. (**a**) Percentage of freezing recorded during the stage preceding the administration of the US and CS (baseline). (**b**) Percentages of freezing during the contextual test 24 h after the administration of the stimuli. (**c**) Time course of the percentage of freezing in 1-min bins during the contextual test. Data are expressed as means ± S.E.M: *P < 0.05, Tukey’s multiple comparisons test.

Next, the experimental cohort was assessed for associative learning and memory in the cued and contextual fear conditioning test at the age of eighteen months. One WT and one *Mapt*^−/−^ died before the start of the experiment. Percentage of freezing during the two minutes preceding the administration of the unconditioned (US) and conditioned (CS) stimuli did not show any significant difference indicating the absence of any spontaneous freezing alteration [F (2, 53) = 2.535, P = 0.0811, Fig. 10a]. As shown in Fig. 10b, control mice exhibited a greater percentage of freezing than *Mapt*^−/−^ during the first two minutes of exposure to the former context [ANOVA, F (2, 53) = 3.904, P = 0.0262]. This result is more evident when the freezing’s percent over the time was analyzed [genotype [F (2, 53) = 1.604; P = 0.2108], time [F (4, 212) = 13.09, P < 0.0001], time × genotype interaction [F (8, 212) = 4.017, P* = *0.0002, Tukey’s] (Fig. 10c). Thus, our results indicate that the deletion of both tau alleles interferes with associative memory. Table 1 summarizes the number and the age of male mice used for each experiment.

**Table 1:** Summary of male mice of each genotype used for each experiment at each age.

*YM*	*6 months*	*12 months*
WT	N = 20	N = 20
*Mapt* ^+/−^	N = 21	N = 21
*Mapt* ^−/−^	N = 17	N = 17
***EZM***	***6 months***	***12 months***
WT	N = 20	N = 20
*Mapt* ^+/−^	N = 21	N = 21
*Mapt* ^−/−^	N = 17	N = 17
***OF***	***7 months***	***12 months***
WT	N = 20	N = 20
*Mapt* ^+/−^	N = 21	N = 21
*Mapt* ^−/−^	N = 17	N = 17
***NOR***	***7 months***	
WT	N = 20	
*Mapt* ^+/−^	N = 21	
*Mapt* ^−/−^	N = 17	
***MWM***	***10–11 months***	
WT	N = 20	
*Mapt* ^+/−^	N = 21	
*Mapt* ^−/−^	N = 17	
***RAWM***	***16 months***	
WT	N = 20	
*Mapt* ^+/−^	N = 21	
*Mapt* ^−/−^	N = 17	
***FC***	***18 months***	
WT	N = 19	
*Mapt* ^+/−^	N = 21	
*Mapt* ^−/−^	N = 16	

YM = two-trial Y maze; EZM = elevated zero maze; OF = open field; NOR = novel object recognition; MWM = Morris water maze; RAWM = radial arm water maze; FC = fear conditioning.

### Long-term potentiation is impaired in *Mapt*^−/−^ mice

**Figure 11:**
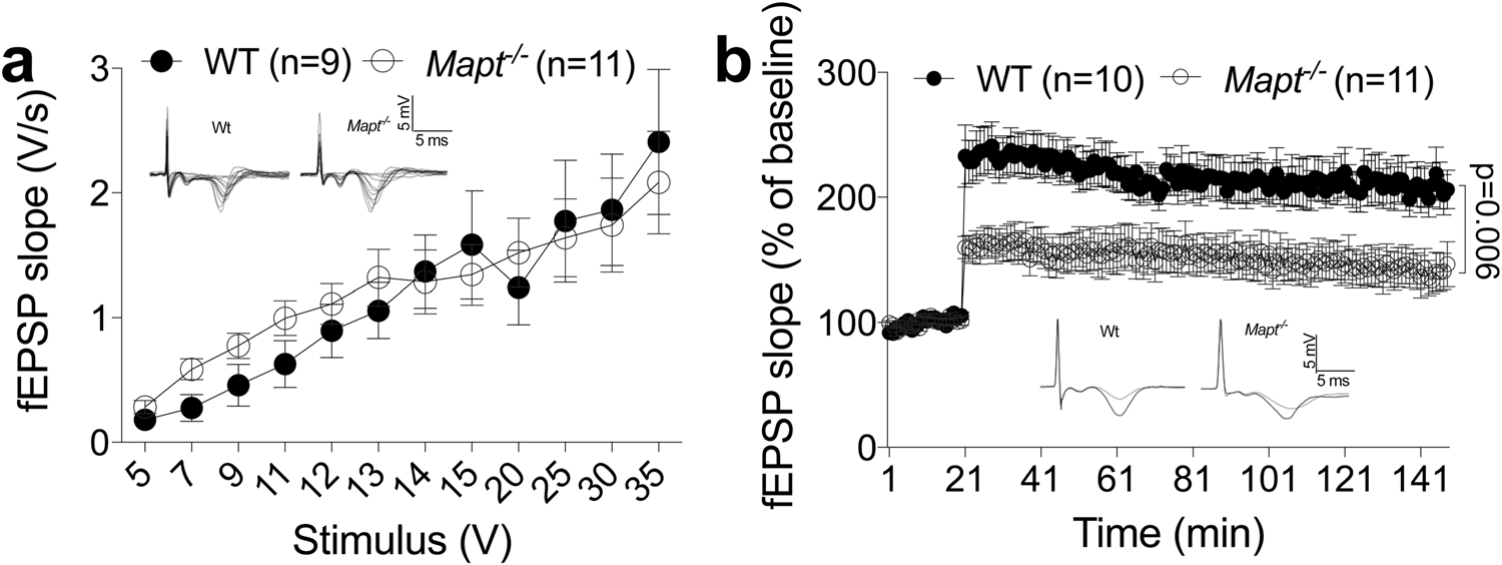
Impaired LTP in old *Mapt*^−/−^ mice. (**a**) Input/output curve: field potentials were recorded from 400 μm hippocampal slices at increasing values of voltage intensity stimulation. Data are expressed as means ± S.E.M. (**b**) Deletion of the *Mapt* gene significantly reduces LTP evoked with theta-burst tetanus at Schaeffer collateral CA3–CA1 synapses. Representative traces 1 min before (thin) and 120 min after (thick) θ-burst stimulation are shown.

**Figure 12:**
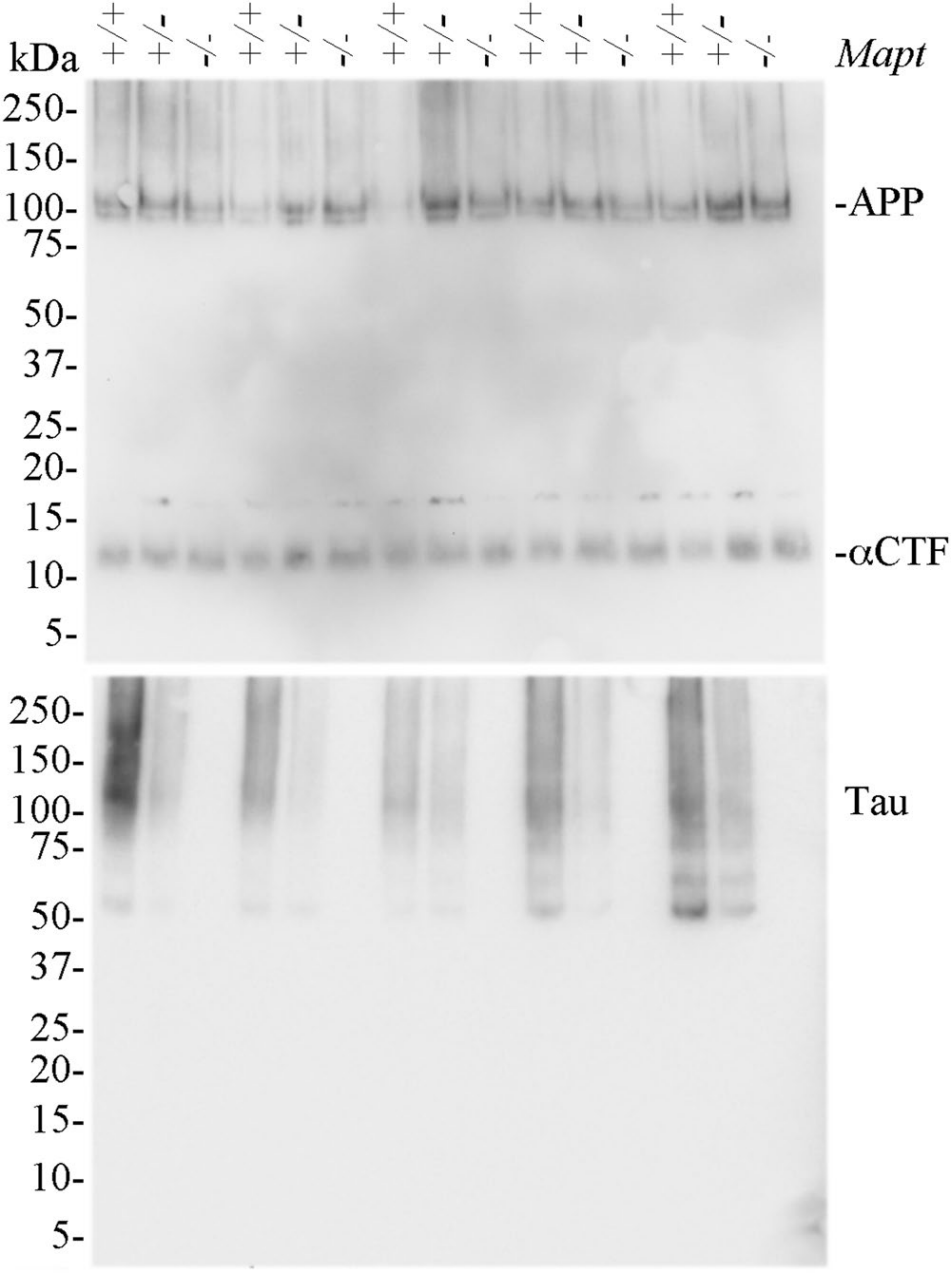
Tau protein is reduced in *Mapt*^+/−^ brains and absent in *Mapt*^−/−^ brains. Western blot analysis of 5 brain homogenates from 5 20-month-old mice for each genotype (*Mapt*^+/+^, i.e. wild type, *Mapt*^+/−^ and *Mapt*^−/−^) rains for with either the anti-APP antibody Y188, which recognize a C-terminal epitope of APP (top panel), of the anti-tau monoclonal antibody DA9 (bottom panel). The data show similar levels of APP and the APP metabolite αCTF in *Mapt*^+/+^, *Mapt*^+/−^ and *Mapt*^−/−^ brains. Yet, levels of tau are highest in *Mapt*^+/+^ samples, reduced in *Mapt*^+/−^ homogenates and ablated in *Mapt*^−/−^ brains. As indicated in the Methods section, the brain samples used for Western blot analysis were prepared from brains of animals perfused under anesthesia with PBS followed by a 4% formaldide solution. This explains why the APP and tau bands appear smeared rather than sharp bands. For this reason, a precise quantitation of signals has not been performed. Nevertheless, it appears clear that the levels of tau are genotype-related and that levels of APP and APP-CTF are, on the contrary, similar in all animals.

Early changes in synaptic function are likely to underlie subtle memory changes in early stages of dementia: thus, given the memory deficits observed in *Mapt*^−/−^ mice, we investigated the role of **t**au in synaptic transmission and plasticity analyzing the Schaffer collateral pathway in hippocampal slices from 18 month old WT and *Mapt*^−/−^ mice. Basal synaptic transmission (BST) was determined by measuring the input-output relationship on the slope of the field excitatory post-synaptic potential (fEPSP) elicited through stimuli of increasing intensity. We found no difference in BST between *Mapt*^−/−^ and WT mice (Fig. 11a) {two-way ANOVA genotype [F (1, 18) = 0.06369, P = 0.8036; two-way ANOVA genotype x intensity interaction [F (11, 198) = 0.8499, P = 0.5905]. LTP, a long-lasting form of synaptic plasticity that is thought to be associated with learning and memory, was markedly impaired in *Mapt*^−/−^ mice as compared to WT littermate controls [ANOVA for repeated measures, F (1, 19) = 9.747; p = 0.006] (Fig. 11b). Our data reflect an overall reduction in LTP of the *Mapt*^−/−^ mice with lower values of potentiation both during the early phase of LTP and late-phase of LTP. The most likely explanation for the observed time course of the potentiation in the *Mapt*^−/−^ mice is that LTP mechanisms underlying different phases of the potentiation are affected. At the end of the experiments 5 animals for each genotype were used to confirm the knockdown and knockout of tau by Western blot analysis. The data (Fig. 12) show that tau expression is reduced in *Mapt*^+/−^ and abolished in *Mapt*^−/−^ mouse brains. Overall, these data are consistent with the cognitive decline caused by *Mapt* deletion.

## Discussion

Given the current therapeutic focus on tau, it is important to better understand the role of tau in normal brain functions, especially functions underlying cognition, learning and memory. In this regard, data in the literature are variable: several studies have reported normal cognition in *Mapt*^−/−^ mice^22,23,42^, while other have uncovered impaired Morris water maze performance, contextual and cued fear condition, the former only in old animals^39,43^.

We have tested *Mapt*^+/−^, *Mapt*^−/−^ and WT littermates on a hybrid B6129PF3/J genetic background. Our analysis has been performed using a *Mapt*^−/−^ line that was employed to generate mice expressing human mutant tau in the absence of murine tau expression^52,53^. The behavioral analysis was started at ~6 months of age. The elevated zero maze and open field tests showed that *Mapt* deletion does not cause anxiety-like behavior (Figs 1 and 3d). These tasks were repeated at 12 months of age with similar results (Figs 6 and 8d). At both ages, *Mapt*^−/−^ mice exhibited a significant greater activity as compared to WT mice in the open field tests (Figs 3a–c and 8a–c). Hyperactivity caused by tau deficiency was previously reported^43,45^. This hyperactivity was not observed in the Y-maze task (Figs 2b and 7b) as also noted by Lei *et al*.^45^, who interpreted these findings with the *Mapt*^−/−^ mice being unable to suppress movement, when there is lack of cognitive intention. *Mapt*^−/−^ mice showed a mild impairment in short-term spatial memory tested in Y-maze task at 6–7 months of age (Fig. 2b), which worsened in an aging-dependent manner (Fig. 7b). A novel object recognition experiment performed at 7 months of age confirmed that *Mapt*^−/−^ mice develop short-term memory deficits (Fig. 4). Interestingly, *Mapt*^+/−^ mice, which performed as well as the WT littermates in the two-trial Y-maze task at both 6 and 12 months of age, presented some deficit in the novel object recognition task.

Consistent with previous reports^42–44^ *Mapt* deletion did not cause spatial reference memory at ten/eleven months of age (Fig. 5). However, *Mapt*^−/−^ mice present deficits in working memory at the age of ~16 months (Fig. 9). This result differs from previous reports, showing that *Mapt*^−/−^ mice presented normal when tested in the radial arm maze^43,54^. Next, we used the fear-conditioning paradigm to test associative memory. In agreement with other published reports^39,43^, *Mapt*^−/−^ mice showed impaired contextual fear conditioning (Fig. 10). However, we cannot exclude that the decreased propensity of *Mapt*^−/−^ mice to freeze in the contextual fear-conditioning test is caused by the increased locomotive activity rather than memory deficits.

Recently, a biological function of tau in dendritic regions and synapses is emerging^22,55,56^. These synaptic functions of tau are emphasized by evidence showing that *Mapt*^−/−^ mice have deficits in LTD^40,41^, a form of synaptic plasticity that underlies the weakening of synaptic connections. In contrast, the role of tau in LTP, which instead potentiates synaptic connections and is thought to underlie learning and memory, is unclear: one report has shown that LTP is not affected by tau deletion^40^ and another had described LTP deficits in *Mapt*^−/−^ mice^39^. Given the memory deficits shown by this cohort of *Mapt*^−/−^ mice, we analyzed LTP in our mice. The data show a reduction in potentiation during both the early and late phase of LTP. Different molecular mechanisms underlie these phases and are responsible for the time course of the potentiation. Albeit interesting, the identification of these molecular mechanisms is beyond the goal of this study. Similarly, the presence of both LTP and LTD deficits in *Mapt*^−/−^ mice stresses the important role of tau in long-term synaptic plasticity^40,41^. Since LTP and LTD have different requirements for their induction, such as persisting low-frequency input to a synapse that does not need to be associated with postsynaptic depolarization in LTD whereas it does for LTP^57^, and yet share some common molecular mechanisms, such as Ca^2+^ entry through NMDA receptors^58^, it is impossible to know, based on the findings of the current manuscript, whether the defect we have observed is due to a mechanism that is different or similar between LTP and LTD.

Although our study does not explain the exact reasons why the analysis of different *Mapt* KO on different genetic background performed in different laboratories have yielded distinct results, it strongly supports the notion that tau plays an important role in learning, memory and long-term plasticity. Our work was conducted using a hybrid mouse model of *Mapt* deletion. The hybrid displays better memory scores than their parental strains (hybrid vigor)^49,50^, which could either facilitate detection of minor memory deficits or, quite the reverse, mask or override subtle effects of *Mapt* deletion or gene dose reduction. At any rate, of the evidence that our mouse model reproduced some of the phenotypes already reported^39,42–44^ supports a role of tau in learning, memory and synaptic plasticity. Moreover, recent data showing that caspase cleavage of tau at Asp^421^ is essential for normal memory and normal LTP^59^ further underline this important role of tau. In future studies it will be important to further test the role of tau in cognition and synaptic plasticity –for example to determine how early LTP is compromised by tau deletion- and to determine whether the effect of ablating tau expression is similar in both sexes. The clear difference in memory deficits between *Mapt*^+/−^ and *Mapt*^−/−^ suggests that therapeutic approaches aiming to reduce toxic tau forms by decreasing tau levels, may have a valuable therapeutic utility in which beneficial effects may occur without the unwanted consequences of excessive tau levels reduction.

## Methods

### Ethics Statement and generation of mice

Mice were handled following the Ethical Guidelines for Treatment of Laboratory Animals of Albert Einstein College of Medicine (AECOM). Animals were housed with a 12-h light/12-h dark cycle in groups of five in plastic cages with ad libitum access to food and water. These procedures followed the Institutional Animal Care and Use Committee (IACUC) guidelines. The behavioral procedures used here were described in detail in animal protocol number 20130509 and were approved by the IACUC at the AECOM. *Mapt*^−/−^ mice (Jackson laboratory, Stock No: 029219) were backcrossed to C57Bl6/J for 10 generations. B6129PF1/J mice were purchased from Jackson laboratory (Stock No: 100492). These F1 hybrid mice are the offspring of a cross between C57BL/6 J females (B6) and 129 P3/J males (129P). *Mapt*^−/−^-C57Bl6/J mice were crossed to B6129PF1/J mice to obtain *Mapt*^+/−^-B6129PF2/J animals. Next, female and males *Mapt*^+/−^-B6129PF2/J were crossed to obtain the experimental animals: *Mapt*^+/−^-B6129PF3/J (referred to as *Mapt*^+/−^), *Mapt*^+/+^-B6129PF3/J (referred to as WT) and *Mapt*^−/−^-B6129PF3/J (referred to as *Mapt*^−/−^). Only male mice have been included in the study to reduce the variability introduced by sex factors such as the hormonal fluctuation that occurs during the estrous cycle^60,61^.

### Behavioral experimental procedures

The investigators were blinded to the group allocation during the entire test. We randomized mice to control for potential litter effects. The sample size was predetermined on the basis of our unpublished data and a recent report testing mice expressing a pathogenic tau mutant protein^62^. Before starting behavioral experiments, each animal was handled for five days. Each experiment was performed during the light cycle in a separate behavioral testing suite after a 30-min-period of acclimation. A software was utilized to track automatically each mouse during all the experiments.

*Elevated Zero Maze* was used to test for anxiety-like behavior as previously described in detail^63,64^. Briefly, each mouse was allowed to explore the enclosed and open arenas of the annular apparatus for 5 min. ANY-maze software was used to track mice’s movements (ANY-maze, Stoelting). Anxiety was assessed as time dwelling in the closed arena, while a longer time dwelling in the open arena was considered as index of lower anxiety.

*Y-maze* was used to test for spatial recognition memory as previously described in detail^63^. Briefly, each mouse was allowed to explore two arms of the Y-maze apparatus during the first trial (training). One hour later, the third arm was opened, and the mouse was returned to the same apparatus and allowed to explore all the three arms (testing). Extramural visual cues were used as tool for environment exploration. The percentage of entries into the arms was calculated as index of exploration of the arms.

*Open Field* was used to test for locomotor, exploratory and anxiety-related behavior as previously described in detail^63^. Each mouse was allowed to explore the open field for 10 min. The indexes of motility (distance, speed, time spent traveling at speed greater than 50 mm/s) and of anxiety (time spent in the center of the field) were obtained using the ANY-maze software. Two daily sessions were conducted to assess the level of habituation to the box previously explored.

#### Novel Object Recognition

The same open field apparatus was used to perform the novel object recognition test to assess visual recognition memory, which is a non-aversive task that relies on rodents’ natural exploratory behavior. During the training session, two identical non-toxic objects were placed in opposite and symmetrical corners of the arena; each mouse was released in the center of the opposite wall facing the wall. Animals were left to explore space and objects for a 10-minutes period. Four hours after this initial exploration, the animals were returned to explore the arena, where one known object was replaced by a novel object, for an additional 10 min. Object exploration was defined as the animal’s nose pointing to the object at a distance of 2 cm or less. The object discrimination ratio was calculated by dividing the time the mice spends exploring the novel object by the total amount of time spent exploring the two objects and an object discriminatory index, which was obtained dividing the difference in exploration time between the novel and familiar objects by the total time spent exploring both objects. To reduce unwanted and unspecific effects related to preference for an object and for the position/side in which the object was placed, the novelty of the objects (i.e., novel vs. familiar) and the location of the novel object (i.e., right vs. left) were balanced within each genotype.

#### Water mazes

Mice were first tested in the visible platform task to evaluate locomotor, motivational or visual defects followed by the Morris water maze and radial arm water maze tests for spatial reference memory and working memory, respectively. Test were performed as previously described in detail^63^. In Morris water maze, the swimming path covered to locate the platform was tracked by the ANY-maze software. Mice were trained in two training sessions of three trials per day. Two days after the first five-day session retention memory was assessed in a single probe trial in which the platform was removed. Two and five days after the second three-day session mice were further assessed for retention memory in two separate probe trials. Time spent in the target quadrant, number of crossings of the area where the platform was originally located, proximity to the same area were calculated and considered as index of reference memory.

In the radial arm water maze protocol, the mean number of errors made by each mouse over the last three experimental days was calculated and analyzed by ANOVA. The experiment was completed when the performance criteria were satisfied (number of errors ≥ 2 on trial 3).

#### Fear Conditioning

Animals were tested for associative learning and memory as previously described in detail^63^. Mice were placed in the conditioning chamber for 2 min before the onset of a discrete tone which comprised the conditioned stimulus (CS); the sound lasted 30 s at 2,800 Hz and 85 dB). In the last 2 s of the CS, mice were given a foot shock, comprising the unconditioned stimulus (US), of 0.60 mA for 2 s through the bars of the floor. After the CS/US pairing, the mice were left in the conditioning chamber for another 30 s and were then placed back in their home cages. Freezing behavior, defined as the absence of all movement except for that necessitated by breathing, was scored using the FreezeFrame software (Coulbourn Instruments). To evaluate contextual fear learning, freezing was measured for 5 min (consecutive) in the chamber in which the mice were tested 24 h after training.

Sensory perception of the shock was determined through threshold assessment after completion of the test. In brief, each animal was shocked by two sequential foot shocks at 0.1, 0.2, 0.4, and 0.6 mV. Shock intensity curve was produced by assigning 5 different scores based on the reaction observed upon the delivery of each shock: 0 = no response, 1 = ambulation, 2 = flinch, 3 = hop, 4 = run, and 5 = jump.

### Electrophysiological Studies

Hippocampal slices (400 μm) were cut with a tissue chopper and maintained in an interface chamber at 29 °C for 90 minutes prior to recording, as previously described^65^. Following assessment of basal synaptic transmission by plotting the stimulus voltages against slopes of field Excitatory Post-Synaptic Potentials (fEPSP); baseline was recorded every minute at an intensity that evoked a response 35% of the maximum evoked response. LTP was induced using a theta-burst stimulation (4 pulses at 100 Hz, with the bursts repeated at 5 Hz and each tetanus including 3 ten-burst trains separated by 15 sec). Responses were measured as fEPSP slopes expressed as percentage of baseline.

### Mouse brain and Western blot analysis

The brain samples used for Western blot analysis were prepared as follows. At the end of behavioral experiments, mice were perfused under anesthesia with PBS followed by a 4% formaldide solution. Brains were removed and hemi-brains were homogenized in 5 mM Hepes/NaOH pH 7.4, 1 mM EDTA, 1 mM EGTA, 0.25 M sucrose supplemented with protease and phosphatase inhibitors (Thermo Scientific). Brain homogenates were centrifuged at 800 g for 10 min. Supernatant was collected and analyzed on Western blot analysis for tau expression (mouse monoclonal antibody DA9, gift of Peter Davies) and APP expression (Y188, Abcam).

### Statistical Analysis

All the experimental data were calculated using Prism software (GraphPad, LaJolla, CA). Two-way ANOVA was selected when two independent variables were considered in the experimental design (i.e. time and genotype in the water maze). One-way ANOVA was carried out when only the genotype was considered as the only independent variable and diverse performance measures such as Time in the Open zone, Distance Traveled, Discriminatory Ratio were considered as dependent variables. When significant effects for genotype or significant genotype x independent variable interaction were detected, the analysis was followed by a *post hoc* multiple comparisons test recommended by the Prism software (Tukey’s, Sidak’s or Dunnett’s). A *p* <0.05 was considered statistically significant.
